# Utility of Fiberoptic Endoscopic Evaluation of Swallowing After Left Ventricular Assist Device Implantation

**DOI:** 10.7759/cureus.42291

**Published:** 2023-07-22

**Authors:** Omar M Sharaf, Kevin A Hao, Daniel S Demos, Emily K Plowman, Mustafa M Ahmed, Eric I Jeng

**Affiliations:** 1 Division of Cardiovascular Surgery, University of Florida Health, Gainesville, USA; 2 Division of Cardiovascular Medicine, University of Florida Health, Gainesville, USA

**Keywords:** dysphagia, fees, fiberoptic endoscopic evaluation of swallowing, lvad, left ventricular assist device

## Abstract

Objective

Dysphagia following cardiac surgery is common and associated with adverse outcomes. Among patients receiving left ventricular assist devices (LVAD), we evaluated the impact of fiberoptic endoscopic evaluation of swallowing (FEES) on outcomes.

Methods

A single-center pilot study was conducted in adults (≥18 years of age) undergoing durable LVAD (February 2019 - January 2020). Six patients were prospectively enrolled, evaluated, and underwent FEES within 72 hours of extubation-they were compared to 12 control patients. Demographic, surgical, and postoperative outcomes were collected. Unpaired two-sided t-tests and Fisher’s exact tests were performed.

Results

Baseline characteristics were similar between groups. Intraoperative criteria including duration of transesophageal echo (314 ± 86 min) and surgery (301 ± 74 min) did not differ. The mean time of intubation was comparable (57.3 vs. 68.7 hours, *p*=0.77). In the entire cohort, 30-day, one-year, two-year, and three-year mortality were 0%, 5.6%, 5.6%, and 16.7%, respectively. Sixty-seven percent of the patients that underwent FEES had inefficient swallowing function. The FEES group trended to a shorter hospital length of stay (LOS) (29.1 vs. 46.6 days, *p*=0.098), post-implantation LOS (25.3 vs 30.7 days, *p*=0.46), and lower incidence of postoperative pneumonia (16.7% vs. 50%, *p*=0.32) and sepsis (0% vs. 33.3%, *p*=0.25).

Conclusion

FEES did not impact 30-day, one-year, two-year, or three-year mortality. Though not statistically significant, patients who underwent FEES trended toward shorter LOS and lower postoperative pneumonia and sepsis rates. Additionally, we report a higher incidence of dysphagia among patients undergoing FEES despite comparable baseline risk factors with controls.

## Introduction

Postoperative aspiration and dysphagia is an increasingly common, yet understudied, complication post cardiac surgery [1-5]. Dysphagia is characterized by impairments in swallowing efficiency (inability to adequately propel food and liquid from the mouth to the stomach) and swallowing safety (inability to protect the airway during swallowing), leading to aspiration of ingested materials into the trachea and pulmonary tract [6]. Consequences of dysphagia following cardiac surgery include malnutrition reintubation, increased hospital length of stay (LOS), and aspiration pneumonia [6]. Although preventable, the latter represents the leading cause of morbidity following open heart surgery [7,8]. Previous studies have suggested that timely diagnosis of dysphagia is critical to prevent devastating aspiration events [9].

The use of flexible endoscopy to evaluate dysphagia, fiberoptic endoscopic evaluation of swallowing (FEES), is a safe, convenient, and effective tool for evaluating dysphagia [10-17]. Notably, when compared to the modified barium swallow (MBS) study, numerous studies have demonstrated that FEES has greater or equal sensitivity to detect key swallowing parameters: delay in swallowing initiation, penetration, aspiration, and pharyngeal residue [18-21]. Additionally, FEES has been suggested to more frequently identify penetration and aspiration compared to MBS [11,20]. Furthermore, FEES is an attractive alternative to conventional video fluoroscopy for evaluating dysphagia due to its portability, ease of use, tolerability, and absence of radiation exposure [10].

We conducted a single-center prospective pilot study to evaluate the impact of FEES on health-related outcomes among patients undergoing durable left ventricular assist device (LVAD) implantation. Specifically, we aimed to assess the incidence of developing dysphagia and related postoperative outcome measures between patients who underwent FEES and a cohort of matched control patients with similar baseline risk factors. This project was presented at the American College of Surgeons Clinical Congress 2021 (Virtual; October 2021). A preprint of the manuscript [22] has previously been published, but this article is not currently under consideration elsewhere.

## Materials and methods

Study population and patient characteristics

The University of Florida Institutional Review Board (IRB) approval was obtained for this single-center study of FEES in adult patients (≥18 years of age and <90 years of age) undergoing durable LVAD placement (IRB #202001240). Patient informed consent was obtained for postoperative FEES. Over one year (February 2019 - January 2020), 25 patients received a durable ventricular assist device at the study institution; patients undergoing biventricular assist device implantation were excluded. Six patients undergoing durable LVAD placement were enrolled prospectively during the study period. These patients underwent FEES within 72 hours of extubation postoperatively. A group of 12 control patients also undergoing durable LVAD placement but not FEES from the same one-year period were evaluated for comparison.

Data collection

Data were collected retrospectively from the electronic medical record. Demographic and baseline data collected include sex, age, body mass index (BMI), race, tobacco use, and past medical history including diabetes mellitus, hypertension, and hyperlipidemia. Predicted operative mortality was calculated for each patient, accounting for patient risk factors, using the European System for Cardiac Operative Risk Evaluation II (EuroSCORE II) [23]. Intraoperative data collected include LVAD device type, transesophageal echocardiography (TEE) time, cardiopulmonary bypass (CPB) time, total operative time, and total intubation time including during the postoperative period. The intent of therapy as a bridge to transplant or destination therapy was also recorded. Total intubation time was recorded for the primary intubation event from the immediate preoperative intubation time to postoperative extubation time.

Dysphagia variables collected include whether a speech-language pathologist (SLP) was consulted, whether a barium swallow study was performed and time to swallow study following surgery, and dysphagia status and severity. Dysphagia status was recorded as a binary variable, and severity was recorded as described in the electronic medical record as mild, moderate, or severe.

Health-related outcomes recorded include total time nil per os [(NPO); nothing by mouth] postoperatively, intensive care unit (ICU), postoperative and total LOS, incidence of pneumonia and sepsis, 30-day readmission, and 30-day, one-year, two-year, and three-year mortality. Total time NPO was defined as the time from surgery to the first diet order by mouth excluding orders for ice chips. Pneumonia was recorded when the medical record documented clinical suspicion, supporting chest x-ray findings and clinical features, and a plan of management.

Statistical analysis

Unpaired two-sided t-tests and Fisher’s exact tests were performed for continuous and categorical variables, respectively. Survivorship between patients undergoing FEES was compared to the control group using the Kaplan-Meier method and the log-rank test. Patients were censored at the date of death or most recent follow-up as appropriate. All statistical analyses were performed using R Software (version 3.6.3; R Core Team, Vienna, Austria) with a defined α = 0.05 [24].

## Results

Demographics

Of the 18 patients (66.7% male) included for analysis, six (33.3%) underwent FEES examination following LVAD surgery (Table 1). Patients undergoing FEES were comparable in age (62 ± 15 vs 54 ± 12 years, P ­­= 0.32) and BMI (32.9 ± 8.2 vs 28.1 ± 4.5, P = 0.23) to the matched control patients. Sex and race were comparable between cohorts (P = 1.00 and P = 0.67, respectively). There were no substantial differences between cohorts in the incidence of active tobacco use, diabetes mellitus, hypertension, and hyperlipidemia. Matched control patients had higher EuroSCORE II values though not statistically significant (6.0 ± 3.2 vs 10.6 ± 6.5, P = 0.06). All patients underwent beating heart on-pump centrifugal intrathoracic LVAD implantation with sternotomy without ischemia, due to decompensated heart failure. All patients received either the HeartMate III (Abbott, Abbott Park, Illinois, USA) or HeartWare (Medtronic, Minneapolis, Minnesota, USA) (P = 0.85). Patients in the FEES cohort did not differ from the matched cohort based on TEE time (325.2 ± 40.4 ­­vs 308.1 ± 103.0 minutes, P = 0.62), CPB time (91.3 ± 26.0 vs 96.1 ± 39.7 minutes, P = 0.71), operative time (308.5 ± 34.3 vs 297.4 ± 88.8 minutes, P = 0.38), and total intubation time (68.7 ± 75.9 vs 57.3 ± 82.3 minutes, P = 0.77).

**Table 1:** Baseline Demographics BMI: body mass index; CPB: cardiopulmonary bypass; EuroSCORE: European System for Cardiac Operative Risk Evaluation; IQR: interquartile range; TEE: Transesophageal Echocardiogram. Values are % (n) unless otherwise indicated. ^a ^Data unavailable for two patients in matched cohort.

Variable	All patients (n = 18)	FEES (n = 6)	Matched (n = 12)	p Value
Preoperative	-----	-----	-----	-----
Age, years	-----	-----	-----	-----
Mean ± SD	57 ± 13	62 ± 15	54 ± 12	0.32
Median (IQR)	59 (48, 68)	67 (59, 71)	56 (47, 64)	-----
Male	66.7 (12)	66.7 (4)	66.7 (8)	1.00
Race	-----	-----	-----	0.67
Asian	0 (0)	0 (0)	0 (0)	-----
Black	33.3 (6)	16.7 (1)	41.7 (5)	-----
Hispanic	5.6 (1)	0 (0)	8.3 (1)	-----
White	27.8 (5)	66.7 (4)	8.3 (1)	-----
Other	33.3 (6)	16.7 (1)	41.7 (5)	-----
BMI, kg/m^2^, mean ± SD	29.7 ± 6.2	32.9 ± 8.2	28.1 ± 4.5	0.23
Tobacco use	0 (0)	0 (0)	0 (0)	-----
Prior stroke	16.6 (3)	16.6 (1)	16.6 (2)	1.00
Previous swallowing disorder	5.56 (1)	0 (0)	8.3 (1)	1.00
Gastroesophageal reflux disease	22.2 (4)	16.6 (1)	25.0 (3)	1.00
Albumin (g/dL), mean ± SD	3.80 ± 0.25	3.80 ± 0.43	3.77 ± 0.26	0.85
Diabetes	27.8 (5)	33.3 (2)	25.0 (3)	1.00
Hypertension	72.2 (13)	50.0 (3)	83.3 (10)	0.27
Hyperlipidemia	66.7 (12)	66.7 (4)	66.7 (8)	1.00
EuroSCORE II, mean ± SD	9.0 ± 5.9	6.0 ± 3.2	10.6 ± 6.5	0.06
INTERMACS Profile, mean ± SD	3.50 ± 0.8	3.83 ± 0.4	3.33 ± 0.9	0.23
Intraoperative	-----	-----	-----	-----
Bridge to transplant	22.2 (4)	0 (0)	33.3 (4)	0.25
Device	-----	-----	-----	0.61
HeartMate III	72.2 (13)	83.3 (5)	66.7 (8)	-----
HeartWare	27.8 (5)	16.7 (1)	33.3 (4)	-----
TEE time, minutes^a^	313.8 ± 86.1	325.2 ± 40.4	308.1 ± 103.0	0.62
CPB time, minutes	94.5 ± 35.0	91.3 ± 26.0	96.1 ± 39.7	0.71
Operative time, minutes	301.1 ± 74.0	308.5 ± 34.3	297.4 ± 88.8	0.38
Total Intubation time, hours	61.1 ± 78.2	68.7 ± 75.9	57.3 ± 82.3	0.77

Dysphagia status

Dysphagia status is shown in Table 2. Postoperative NPO time trended higher in the cohort undergoing FEES but did not differ significantly between groups (175.2 ± 185.3 ­­vs 50.5 ± 67.7 hours, P = 0.16). Half of the LVAD patients were consulted by an SLP. While SLP consultation was similar between FEES and matched control cohorts (66.7% vs 41.7%, P = 0.62), all four patients who underwent a barium swallow study were in the FEES cohort (66.7% vs 0%, P = 0.005). Time to swallow trended towards shorter times in the FEES group (8.0 ± 6.2 vs 28.4 ± 14.5 days, P = 0.06). The detected incidence of dysphagia was significantly greater in the FEES cohort compared to matched controls (66.7% vs 0%, P < 0.001). Of the six patients that underwent FEES, four were determined to be dysphagic; severity ranged from mild (N = 2) to moderate (N = 1) to severe (N = 1). Dysphagia status and severity were not noted in any of the matched control patients, as these statuses were dependent on either FEES or MBS.

**Table 2:** Dysphagia Status NPO: nil per os (nothing by mouth); SLP: Speech-Language Pathologist. Values are % (n) unless otherwise indicated. ^a ^Reported for 4 patients in each cohort.

Variable	All patients (n = 18)	FEES (n = 6)	Matched (n = 12)	p Value
NPO time, hours	92.1 ± 129.3	175.2 ± 185.3	50.5 ± 67.7	0.16
SLP Consultation	50.0 (9)	66.7 (4)	41.7 (5)	0.62
Study Performed	-----	-----	-----	-----
Barium Swallow	22.2 (4)	66.7 (4)	0 (0)	0.005
Time to Swallow^a^, days	18.2 ± 15.0	8.0 ± 6.2	28.4 ± 14.5	0.06
Dysphagia Status	-----	-----	-----	< 0.001
Not Noted	66.7 (12)	0 (0)	12 (100)	-----
Within Normal Limit	11.1 (2)	33.3 (2)	0 (0)	-----
Dysphagic	22.2 (4)	66.7 (4)	0 (0)	-----
Dysphagia Severity	-----	-----	-----	0.005
Mild	11.1 (2)	33.3 (2)	0 (0)	-----
Moderate	5.6 (1)	16.7 (1)	0 (0)	-----
Severity	5.6 (1)	16.7 (1)	0 (0)	-----

Health-related outcomes

**Figure 1:**
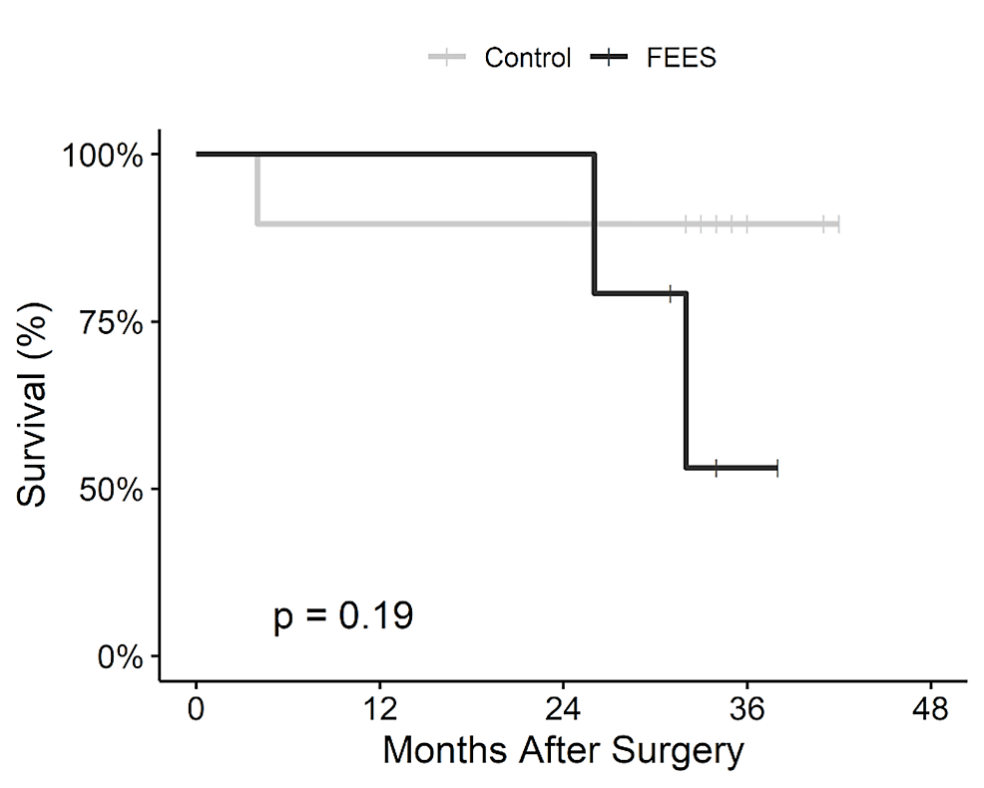
Kaplan-Meier Survivorship for Patients Undergoing FEES Compared to Matched Controls with Log-Rank Test FEES: fiberoptic endoscopic evaluation of swallowing.

Patients in the FEES cohort did not significantly differ based on cardiac ICU (CICU) LOS (10.9 ± 7.3 vs 12.1 ± 5.1 days, P = 0.72), postoperative LOS (25.3 ± 13.2 vs 30.7 ± 15.4 days, P = 0.46), and total LOS (29.1 ± 12.9 vs 46.6 ± 29.4 days, P = 0.10) (Table 3). Patients who underwent FEES trended towards a lower incidence of postoperative pneumonia (16.7% vs 50.0%, P = 0.32) and postoperative sepsis (0% vs 33.3%, P = 0.25). One patient in each cohort was readmitted within 30 days; however, 30-day and one-year mortality were 0% and 5.6%, respectively, for the entire cohort. Mortality at two and three years and Kaplan-Meier survivorship did not differ between groups (Figure 1).

**Table 3:** Health-Related Outcomes CICU: Cardiac Intensive Care Unit; LOS: length of stay; NPO: not by mouth. Values are % (n) unless otherwise indicated.

Variable	All patients (n = 18)	FEES (n = 6)	Matched (n = 12)	p Value
CICU LOS, days	11.7 ± 5.7	10.9 ± 7.3	12.1 ± 5.1	0.72
Postoperative LOS, days	28.9 ± 14.5	25.3 ± 13.2	30.7 ± 15.4	0.46
Total LOS, days	40.8 ± 26.1	29.1 ± 12.9	46.6 ± 29.4	0.10
NPO time, hours	92.1 ± 129.3	175.2 ± 185.3	50.5 ± 67.7	0.16
Pneumonia	38.9 (7)	16.7 (1)	50.0 (6)	0.32
Sepsis	22.2 (4)	0 (0)	33.3 (4)	0.25
30-day readmission	11.1 (2)	16.7 (1)	8.3 (1)	1.00
30-day mortality	0 (0)	0 (0)	0 (0)	-----
1-year mortality	5.6 (1)	0 (0)	8.3 (1)	1.00
2-year mortality	5.6 (1)	0 (0)	8.3 (1)	1.00
3-year mortality	16.7 (3)	33.3 (2)	8.3 (1)	0.25

## Discussion

Despite the increasing popularization of the use of FEES, its efficacy specifically in the setting of cardiac surgery has not been well studied and consequently is seldomly used [11]. In a 2021 study, tracheal aspiration was found to be prevalent, costly to patients, and associated with increased morbidity and mortality after adult cardiac surgery [25]. Given the high incidence of dysphagia and concurrent silent aspiration and subsequent pneumonia in cardiac surgery patients, demonstration of the efficacy of FEES in the setting of cardiac surgery could play an important role in increasing its utilization and improving health-related outcomes [26].

Postoperative dysphagia following cardiac surgery is common, multifactorial, and is associated with increased morbidity and increased LOS [3,6]. FEES is a convenient tool for evaluating dysphagia and has been shown to decrease the incidence of aspiration pneumonia in other settings, but its use in postoperative cardiac surgical care has not been adopted as a standard of care [10,11]. In our review of patients undergoing durable LVAD implantation, patients that underwent FEES trended towards shorter total hospital and post-implant LOS and lower postoperative pneumonia and sepsis rates. In the entire LVAD cohort, we had zero 30-day, and 5.6% one-year mortality, so FEES intervention did not impact mortality. Mortality at two and three years was 5.6% and 16.7%, respectively.

Dysphagia is a significant complication post-cardiac surgery that requires attention and mitigation. Risk factors for developing dysphagia following cardiac surgery include TEE use, prolonged operative duration, prolonged mechanical ventilation, New York Heart Association classes III and IV, and larger endotracheal tube size [1,2,4,5,9,25,27,28]. TEE use was identified as an independent predictor of dysphagia among 869 patients undergoing cardiac surgery [5]. In another study of operative duration and dysphagia among 838 patients undergoing cardiac surgery, no patients who were operated on for less than 4.5 hours developed dysphagia, suggesting an association exists between operative duration and dysphagia [4]. Studies have also identified prolonged mechanical ventilation as a risk factor for dysphagia among patients undergoing cardiac surgery [1,2,4,5,9,27].

Identifying dysphagia in patients early in the postoperative period may also contribute to reduced adverse outcomes associated with dysphagia. Though prior studies demonstrate FEES use in adult and pediatric settings outside of cardiac surgical care is safe and effective for evaluating dysphagia [10-17], it has not been until recently that studies have incorporated FEES to detect dysphagia in cardiac surgery [29]. In a prospective trial of adult patients undergoing elective cardiac surgery, FEES was used as confirmatory testing for patients failing a targeted swallow screen; the study found the true incidence of dysphagia after cardiac surgery to be significantly higher than previously recognized [25,28,30]. In a study of 60 patients with dysphagia of various origins, FEES had high sensitivity and validity for the detection of dysphagia [31]. Although the study was not limited to patients undergoing cardiac surgery, the finding that FEES has high sensitivity and validity for detecting dysphagia may explain the higher incidence of dysphagia noted among LVAD patients undergoing FEES in the current study despite comparable baseline risk factors with the control group. These findings suggest that dysphagia may be underdiagnosed without instrumentation, and early subclinical dysphagia can be detected with FEES. However, it is important to note that not all hospital ecosystems have FEES capabilities, but other means such as conventional video fluoroscopy and MBS are still suitable alternatives.

We believe that while our pilot study importantly contributes to understanding how FEES can be integrated into LVAD surgery postoperative care, it is limited due to its retrospective nature, small sample size, and low event rates, limiting our ability to draw conclusions that can be generalized beyond the patients in the present study. Therefore, we evaluated categorical variables by Fisher’s exact test and restricted our conclusions to preliminary findings requiring further study.

## Conclusions

Dysphagia following durable LVAD implantation is common and its consequences may be preventable through early detection utilizing FEES. Our preliminary findings suggest that postoperative FEES use is safe in the setting of LVAD implantation. Despite comparable baseline cited risk factors for dysphagia between the FEES group and non-FEES group, LVAD patients in the FEES group had a higher incidence of dysphagia, suggesting FEES may have high sensitivity for detecting subclinical dysphagia. This may translate to heightened clinical vigilance and trends toward reduced adverse clinical outcomes following durable LVAD implantation; however, a larger prospective study is warranted to delineate the significance of these preliminary findings.
